# Editorial: Neurological and neuropsychiatric disorders affecting military personnel and veterans

**DOI:** 10.3389/fneur.2024.1392721

**Published:** 2024-03-12

**Authors:** Chen Lin, Mary Jo Pugh, Venkatagiri Krishnamurthy, Lisa C. Krishnamurthy, Willliam C. Walker

**Affiliations:** ^1^Department of Neurology, University of Alabama at Birmingham, Birmingham, AL, United States; ^2^Birmingham VA Medical Center, Birmingham, AL, United States; ^3^Department of Medicine, University of Utah, Salt Lake City, UT, United States; ^4^Salt Lake City VA Medical Center, Salt Lake City, UT, United States; ^5^Atlanta VA Medical Center, Decatur, GA, United States; ^6^Department of Neurology, Emory University, Atlanta, GA, United States; ^7^Department of Physics and Astronomy, Georgia State University, Atlanta, GA, United States; ^8^Joint GSU, Georgia Tech, and Emory Center for Translational Research in Neuroimaging and Data Science (TReNDS), Atlanta, GA, United States; ^9^Department of Radiology and Imaging Sciences, Emory University, Atlanta, GA, United States; ^10^Department of Physical Medicine and Rehabilitation (PM&R), School of Medicine, Virginia Commonwealth University, Richmond, VA, United States; ^11^Richmond Veterans Affairs (VA) Medical Center, Central Virginia VA Health Care System, Richmond, VA, United States

**Keywords:** veteran affairs, military, neuropsychaitric disorders, neurological and psychiatric symptoms, neurotrauma and neurodegenerative disease, traumatic brain injury (TBI), veteran

Active Service Members (SM) and Veterans of the military face unique neurologic and neuropsychiatric challenges unique to this population compared to the public. SMs and Veteran populations have faced traumatic experiences that lead to both physical and mental consequences. Amongst the important challenges unique to this population include traumatic brain injury (TBI), increased risk of neurological disorders such as dementia and stroke, and comorbid neuropsychiatric conditions. Unfortunately, many of these challenges also have a negative feedback loop such as brain injuries leading to post-traumatic stress disorder (PTSD), which can increase risk of Alzheimer's Dementia. Currently, there are many gaps in the diagnosis, prevention, and treatment of conditions that affect this population disproportionately. There are many opportunities to improve our understanding of these challenges that SMs and Veterans face. The goal of this Research Topic was to shine a light and improve understanding of these challenges. We aimed to collect knowledge from a global network of researchers working on this special Research Topic.

## Neurotrauma

Behavioral dyscontrol is a common sequela of TBI, even when severity is mild (mTBI), and can lead to community reintegration problems and increase risk for suicide. Stromberg et al. examined associations among PTSD symptom severity, deployment-related history of mTBI, and behavioral dyscontrol among SMs and Veterans. Findings showed that SMs and Veterans with PTSD and reduced social support systems are at greatest risk for behavioral dyscontrol. Higher self-efficacy was found to have a protective effect. Findings inform clinical screening strategies and show the need to monitor for behavioral dyscontrol difficulties after mild TBI even in the chronic stages.

The relationship between TBI, its acute and chronic symptoms, and the potential for remote neurodegenerative disease is a priority for military research. Structural and functional connectivity (FC) of the basal ganglia, involved in walking, are altered after TBI. Newsome et al. measured the FC from caudate and pallidum in SMs and Veterans with a history of deployment-related mTBI and their gait. When evaluating the association between FC from the caudate and gait, the non-deployment mTBI group showed a significant positive relationship between walking time and FC with the frontal pole, implicated in navigational planning. Their findings have implications for elucidating subtle motor disruption in SMs and Veterans with mTBI.

van der Veen et al. studied the influence of mTBI history on the relation between balance, gait and sensory function among Veterans and SMs with combat exposure. When sensory systems (vision, vestibular or proprioception) were compromised, the number of mTBIs sustained were associated with lower scores on the Computerized Dynamic Posturography balance assessment. Their findings indicate that processing of sensory information from the vision, proprioception, or vestibular systems were affected long-term after mTBI which in turn affects balance negatively.

After a TBI, the electroencephalogram (EEG) recordings are altered and remain disordered even years after the injury. However, it is still unclear how the changes in EEG recordings relate to cognitive difficulties experienced after TBI. Franke et al. studied 340 service members and veterans, EEG recordings taken 11 years after TBI showed that power in beta and alpha frequencies reflects both injury characteristics and cognitive difficulties, while power in delta frequencies is related to cognitive functions and psychological distress associated with poor long-term outcomes after mTBI.

TBI in Post-9/11 Veterans is concerning due to known associations between TBI and dementia. To avoid potential symptom over-reporting, researchers have used the Validity-10 metric to evaluate for symptom over-reporting. Because the Validity-10 was normed on a relatively healthy sample of young men on active duty, Swan et al. used patient self-report data from the VA comprehensive TBI evaluation to identify characteristics of Veterans with symptom validity failure. The primary factors associated with symptom validity failure were multi-morbidity and polypharmacy of medications affecting the central nervous system. Their findings suggest that use of the Validity-10 to exclude participants from research may be overly restrictive in populations with multimorbidity.

## Dementia and neurodegenerative diseases

There is a growing use of cannabis to self-medicate many symptoms associated with TBI such as chronic pain, headache, insomnia, and cannabinoids may regulate some processes associated with neurodegeneration, Esmaeili, Dismuke-Greer et al. examined the association of cannabis use disorder (CUD) and subsequent diagnosis of diagnoses suggesting cognitive dysfunction. This population-based study of over 1.5 million Veterans found that cognitive disorders was most common among those with CUD and TBI. However those with TBI or CUD alone were also at elevated hazard cognitive dysfunction. Esmaeili, Pogada et al. also examined costs of care for Veterans with different constellations of CUD and cognitive dysfunction. Costs of care in the first 5 years after the TBI index date suggests that costs for those with CUD and dementia tend to emerge over time. Their findings suggest that those with dementia and CUD don't receive care early but that costs of care gradually accumulate over time.

Diagnosis of frontotemporal lobe disorders (FTD) is frequently delayed due to symptoms that are common in other conditions especially behavioral disorders. Hoffman et al. examined a case series of veterans with cognitive and behavioral disorders using epidemiological, clinical, cognitive, laboratory and radiological data. Using this multimodal approach to phenotyping, the authors found distinct symptoms based on etiology for the three primary FTD presentations including 16 different subsyndromes that were characterized by initial overriding and presenting symptoms/syndromes. These distinct subgroups likely require different treatments and inform future research focusted on treating these subsyndromes. Panahi et al. used a population-based approach and natural language processing to identify subsyndromes of FTD in Post-9/11 veterans. Their approach identified a variant with a mix of language and behavioral symptoms which is not typical of FTD presentations. This study suggests that FTD presentation also has a continuum of severity of symptom distress not only across variants but also within variants and may help in identifying FTD early.

Amyotrophic Lateral Sclerosis (ALS) is a progressive neurodegenerative disease that affects the Veterans at a greater rate compared to their civilian counterpart. Kudritzki and Howard reviewed critical aspects of advancing disease-modifying therapies for ALS. The authors also layered the rationale and feasibility of interventions that can potentially be integrated into Veteran Healthcare Services.

## Pain syndromes

The association between headache and remote mTBI is not well established, and risk factors are understudied. Walker et al. demonstrated that headache is extremely common among formerly combat-exposed military personnel and that their remote mTBI history is associated with elevated odds of headache. Blast-related mTBIs were uniquely associated with a higher degree of headache impact on daily life. The findings highlight the ramifications of lifetime mTBI history on headache conditions in the military population.

Non-invasive brain stimulation is gaining traction as a viable tratment approach for neurological and psychiatric conditions. Charvet et al. devised a clinical trial to examine whether at home remotely supervised transcranial direct current stimulation (RS-tDCS) can alleviate the severity and number of headache days in Veterans with persistent post-traumatic headache. The RS-tDCS intervention showed significant decrease in the severity and number of headache days along with high adherence rate. Their results are promising to further advance the utility of tDCS in TBI clinics.

Giakas et al. report the first systematic review and meta-analysis connecting suicidal behavior to chronic pain conditions. The meta-analysis showed that suicidal behavior is greatest in patients with migraine and significantly elevated in back/neck pain compared to a non-pain control group. The elevated risk of suicidal behavior in both migraine and neck/back pain patients underscores the critical need for suicide prevention in Veterans that experience chronic pain.

## Sleep disorders and other conditions

Sleep is a critical pillar of health which is critical to performance of military service members. Rawcliffe et al. examined sleep patterns with wearable technology and self-reported sleep satisfaction in two cohorts of British Army recruits during basic training. The majority of recruits (over 80%) reported poor sleep quality. This led to interference with performance including daytime sleepiness which may impair both cognitive and physical functioning.

Detailed cognitive testing is rarely performed in clinical trials for multiple sclerosis (MS). Relating clinically meaningful measures of MS disability to easily obtainable MRI metrics of atrophy in progressive MS populations is important for clinical research and clinical care. This study (Spain et al.) details cross-sectional structure- cognitive function relationships in a large and well-characterized progressive MS Veteran population, and further compares those relationships between secondary and primary progressive MS.

As is evident by these articles, there is complex comorbidity in veterans with neurological and neuropsychiatric conditions. Caring for individuals with complex comorbidity is frequently associated with significant burden and stress. Rattray et al., used qualitative interviews including self-report measures to examine the impact of caring for Veterans recently separated from active military service over 2 years. This study suggest that when caring for Veterans with “invisible” injuries, systems that provide support to these unpaid care partners would benefit the health and wellbeing of both the care partner and the veteran.

## Conclusion

Military personnel and Veterans suffer from unique conditions and thus require unique care. The articles that comprise this special Research Topic highlight the importance of collaborative efforts needed between healthcare professionals, researchers, caregivers, and funding agencies to support the care of this important population. We hope this Research Topic demonstrates the unique challenges that military personnel and Veterans face but also offers insight into the future potential for advancements in their care.

## Author contributions

CL: Conceptualization, Data curation, Formal analysis, Funding acquisition, Investigation, Methodology, Project administration, Resources, Software, Supervision, Validation, Visualization, Writing—original draft, Writing—review & editing. MP: Writing—original draft, Writing—review & editing. VK: Writing—original draft, Writing—review & editing. LK: Writing—original draft, Writing—review & editing. WW: Writing—original draft, Writing—review & editing.

